# Ionic Mechanism Underlying Rebound Depolarization in Medial Prefrontal Cortex Pyramidal Neurons

**DOI:** 10.3389/fncel.2018.00093

**Published:** 2018-04-23

**Authors:** Przemysław Kurowski, Katarzyna Grzelka, Paweł Szulczyk

**Affiliations:** Laboratory of Physiology and Pathophysiology, Center for Preclinical Research and Technology, The Medical University of Warsaw, Warsaw, Poland

**Keywords:** amyloid β-peptide (1–42), BK channels, Ca^++^ channels, Nav1.9 channels, prefrontal cortex, pyramidal neurons, rats, rebound depolarization

## Abstract

Rebound depolarization (RD) occurs after membrane hyperpolarization and converts an arriving inhibitory signal into cell excitation. The purpose of our study was to clarify the ionic mechanism of RD in synaptically isolated layer V medial prefrontal cortex (mPFC) pyramidal neurons in slices obtained from 58- to 62-day-old male rats. The RD was evoked after a step hyperpolarization below −80 mV, longer than 150 ms in 192 of 211 (91%) tested neurons. The amplitude of RD was 30.6 ± 1.2 mV above the resting membrane potential (−67.9 ± 0.95 mV), and it lasted a few 100 ms (*n* = 192). RD could be observed only after preventing BK channel activation, which was attained either by using paxilline, by removal of Ca^++^ from the extra- or intracellular solution, by blockade of Ca^++^ channels or during protein kinase C (PKC) activation. RD was resistant to tetrodotoxin (TTX) and was abolished after the removal of Na^+^ from the extracellular solution or application of an anti-Nav1.9 antibody to the cell interior. We conclude that two membrane currents are concomitantly activated after the step hyperpolarization in the tested neurons: a. a low-threshold, TTX-resistant, Na^+^ current that evokes RD; and b. an outward K^+^ current through BK channels that opposes Na^+^-dependent depolarization. The obtained results also suggest that a. low-level Ca^++^ in the external medium attained upon intense neuronal activity may facilitate the formation of RD and seizures; and b. RD can be evoked during the activation of PKC, which is an effector of a number of transduction pathways.

## Introduction

The prefrontal cortex fulfills important cognitive functions, including mental set-shifting, inhibition, working memory, information updating, response monitoring and temporal coding (Szczepanski and Knight, 2014). Damage to the prefrontal function has been implicated in widespread illnesses, such as anxiety and depression (Albert et al., 2014), schizophrenia (Schubert et al., 2015), addiction (Volkow et al., 2016) and senile dementia (Kirshner, 2014). These neuropsychiatric disorders are likely to be caused by abnormal function or prefrontal cortex neurons and prefrontal cortex neural circuits.

Rebound depolarization (RD) is the membrane depolarization that occurs following hyperpolarization in neurons. If RD exceeds a threshold of voltage-dependent Na^+^ currents, rebound excitation, a series of action potentials at RD peak, is evoked (Grenier et al., 1998; Tennigkeit et al., 1998; Fan et al., 2000; Sivaramakrishnan and Oliver, 2001; Timofeev et al., 2002; O’Donnell, 2003; Zheng and Raman, 2009; Boehme et al., 2011). RD is thought to be a mechanism responsible for converting an arriving inhibitory signal into cell excitation, which is subsequently synaptically transmitted to other neurons (Sanchez-Vives and McCormick, 2000). The cell hyperpolarization followed by RD can be elicited synaptically, such as by augmented GABAergic inhibitory synaptic input (Yu et al., 2004) or by hyperpolarizing current injection into the cell (Sanchez-Vives and McCormick, 2000).

RD depends primarily on the intrinsic properties of the neuron and relies on hyperpolarization-dependent de-inactivation or activation of channel currents. Numerous mechanisms, including the de-inactivation of T-type Ca^++^ channels (Boehme et al., 2011) or persistent Na^+^ channels (Sangrey and Jaeger, 2010), the increased availability of Na^+^ (Aman and Raman, 2007) or high-threshold Ca^++^ (Zheng and Raman, 2009) channels, and the activation of hyperpolarization-activated cyclic nucleotide-gated (HCN) channels (Van Hook and Berson, 2010), have been proposed to mediate RD. The possibility that two or more channels are responsible for RD cannot be excluded (Engbers et al., 2013a).

RD has been implicated in the rhythmic discharges and oscillations that occur in neurons, such as during sleep (Wang et al., 1995), locomotor activity (Li and Moult, 2012), and epilepsy (Timofeev et al., 1998; Surges et al., 2006). RD can be recorded in various types of cells neurons, including deep cerebellar nuclei (Boehme et al., 2011), thalamic (Lüthi et al., 1998) and hippocampal (Surges et al., 2006). Hyperpolarization followed by RD has been suggested to facilitate the presence of a bistable behavior in medial prefrontal cortex (mPFC) pyramidal neurons, in which the membrane potential shifts from a down-state (negative membrane potential) to an up-state (positive membrane potential; Gulledge and Jaffe, 1998; Valenti and Grace, 2009; Marzo et al., 2014). Up-states in mPFC pyramidal neurons appear as prolonged depolarizations with persistent activity at the depolarization peak (Branchereau et al., 1996; Shu et al., 2003). Up- and down-states are also widely observed in cortical neurons (Harris and Thiele, 2011; Neske, 2016).

The purpose of our study was to determine the intrinsic mechanism responsible for RD in synaptically isolated layer V mPFC pyramidal neurons.

## Materials and Methods

The study was performed in accordance with the guidelines for the care and handling of laboratory animals in Directive 2010/63/EU of the European Parliament and of the Council and adhered to the national (Official Journal of Laws of 2015, item 266) and local (First Local Ethics Committee for Animal Experimentation in Warsaw) guidelines for the ethical use of animals. The Local Commission for the Well-Being of Animals in Warsaw monitored the experiments (Official Journal of The European Union on 20.10.2010, L276/72 (EN) ANNEX IV, Methods of killing animals, Table 3, states that decapitation is allowed in rodents to kill animals for scientific purposes).

### Slice Preparation

The experiments were performed on the neurons of 72 adult (58–62 days old) male Wistar rats obtained from a local animal facility. The applied experimental procedures were similar to those used in our recent studies (Kurowski et al., 2015; Szulczyk, 2016; Gawlak et al., 2017; Grzelka et al., 2017). Rats were decapitated, and their brains were removed and placed in cold (0–4°C) extracellular solution containing the following (in mM): 125 NaCl, 25 NaHCO_3_, 3 KCl, 1.25 NaH_2_PO_4_, 0.5 CaCl_2_, 6 MgCl_2_, and 25 glucose (bubbled with 95% O_2_/5% CO_2_). Coronal slices (300-μm thick) containing the prefrontal cortex were prepared using a vibratome (Vibratome Line, Leica VT1200S, Nussloch, Germany). The slices were incubated for 7 min in warm (34°C) extracellular solution containing the following (in mM): 125 NaCl, 25 NaHCO_3_, 3 KCl, 1.25 NaH_2_PO_4_, 1 CaCl_2_, 1 MgCl_2_ and 25 glucose (bubbled with 95% O_2_/5% CO_2_). The osmolality was 320–330 mOsm/kg H_2_O and was adjusted with glucose. Then, the slices were incubated at room temperature in the same extracellular solution for at least 60 min before being transferred to the recording chamber.

### Patch-Clamp Recordings

The slices were transferred to a bath chamber (RC-24E, Warner Instruments, LLC, Hamden, MA, USA) on the stage of an upright microscope (BX51WI, Olympus Corporation, Tokyo, Japan). During recording, the slices were perfused with warm (34°C) extracellular solution containing the following (in mM): 125 NaCl, 25 NaHCO_3_, 3 KCl, 1.25 NaH_2_PO_4_, 1 CaCl_2_, 1 MgCl_2_, and 25 glucose (bubbled with 95% O_2_/5% CO_2_). In some cases, the NaCl in the extracellular solution was replaced with an equimolar concentration of choline-Cl (125 mM) or LiCl (125 mM). When a nonselective Ca^++^ channel blocker was applied (Cd^++^), NaH_2_PO_4_ was removed from the extracellular solution. In several experiments, Ca^++^ was removed from the extracellular solution. In some cases, the Ca^++^ concentration in the bath was 0.1 or 0.3 mM, as indicated in the text. To eliminate synaptic inputs, the extracellular solution was supplemented with blockers of glutamatergic (50 μM, DL-2-amino-5-phosphonopentanoic acid, DL-AP5; 10 μM, 6,7-dinitroquinoxaline-2,3-dione, DNQX) or GABAergic transmission (50 μM, picrotoxin) and a blocker of voltage-dependent Na^+^ currents (0.5 μM, tetrodotoxin, TTX).

Recordings were obtained from pyramidal neurons located 600–800 μm from the cortical surface. This area of the mPFC corresponds to layer V pyramidal neurons in 58- to 62-day-old rats (Gawlak et al., 2017). The neurons were observed via differential interference contrast microscopy using a 40× water-immersion objective, a camera (C7500-51), and a camera controller (C2741-62) from Hamamatsu Photonics K.K. (Hamamatsu City, Japan). The neurons chosen for the recordings had a triangular body shape and a characteristic apical dendrite. The recordings were obtained from 211 neurons located in 211 slices.

The current-clamp recordings were obtained in a whole-cell configuration using a Multiclamp 700B amplifier, a Digidata 1440A digitizer, and pClamp 10.6 software (Molecular Devices, Sunnyvale, CA, USA). The pipettes were filled with intracellular solution containing the following (in mM): 110 potassium gluconate, 20 KCl, 0.5 EGTA, 2 MgCl_2_, 2 Na_2_-ATP, 0.4 GTP, 10 HEPES, and 5 NaCl (pH 7.4, osmolality 280 mOsm/kg H_2_O). In some experiments, the pipette solution also contained normal guinea pig IgG (4 μg/ml), an antibody against Nav1.9 channels (4 μg/ml), the chelator BAPTA (100 μM) or EGTA (10 mM), or an amyloid β-peptide (1–42) (Aβ_1–42_, 10 μM). In some experiments, 20 mM of KCl was replaced with 20 mM of KF in the pipette solution.

The open-tip pipette resistance was 4–5 MΩ. The pipette offset potential was zeroed when the pipette tip was dipped into the bath solution. Bridge balance and capacitance neutralization were carefully adjusted before and after every experimental protocol using the utility of the amplifier. After giga-seal formation, the cell membrane was disrupted by suction, and the membrane potential was recorded. The pipettes were formed from borosilicate glass with a filament (O.D.: 1.5 mm, I.D.: 0.86 mm; Sutter Instrument, Novato, CA, USA) using a P-1000 puller (Sutter Instrument, Novato, CA, USA). The membrane potential recordings were low-pass filtered at 1–3 kHz and were digitized at a sampling rate of 10 kHz.

Hyperpolarizing current steps (–20 and −40 pA, 200 ms) were used to estimate passive properties, such as input resistance (*R*_in_), series resistance (*R*_s_), and cell membrane capacitance (C), of the tested pyramidal neurons. *R*_in_ and *R*_s_ were calculated as the slope of the linear fit of the current–voltage (I–V) relationship of either the steady-state current (*R*_in_) or the instantaneous current (*R*_s_). The time constant of the current relaxation (*τ*_m_) was estimated with a mono-exponential fit of the voltage response after a current step of −40 pA. Cell capacitance was calculated from *R*_s_ and *τ*_m_ according to the equation given by Marty and Neher (1995).

### Chemical Compounds

Most of the chemical compounds were purchased from Tocris Bioscience (Bristol, UK). ZD 7288, isradipine, phorbol 12-myristate 13-acetate (PMA), and DNQX were purchased from Hello Bio (Bristol, UK); BAPTA, Cd^++^ and 4α-phorbol 12-myristate 13-acetate (4α-PMA) were purchased from Sigma-Aldrich (St. Louis, MO, USA); TTX was purchased from Latoxan (Valence, France); NNC 55-0396 dihydrochloride (NNC 55-0396), DL-AP5, and anti-Nav1.9 antibody were purchased from Alomone Labs (Jerusalem, Israel); Aβ_1–42_ was purchased from Abcam (Cambridge, UK); and normal IgG was purchased from Santa Cruz Biotechnology (Heidelberg, Germany).

Picrotoxin, DNQX, paxilline, isradipine, NPS 2143, PMA and 4α-PMA were dissolved in DMSO. The final concentration of DMSO in the extracellular solutions containing these compounds was 0.01%. In the experiments using compounds that were dissolved in DMSO, the control extracellular solution also contained DMSO at a concentration of 0.01%. The remaining compounds were dissolved directly in the extracellular solution.

The experiments with PMA, 4α-PMA and isradipine were performed in the dark.

Compounds were applied by dissolving them to the final concentration in the artificial cerebrospinal fluid (ACSF) and were added to the bath (VC-6 six-channel valve controller; Warner Instruments, LLC, Hamden, MA, USA) or to the pipette solution when indicated. One biologically active substance was tested only once on one pyramidal neuron.

### Statistics

The data were analyzed using GraphPad Prism 7 (GraphPad Software, Inc., La Jolla, CA, USA). All results presented throughout the article and in the figures are shown as the means ± SE. Unless otherwise indicated, paired Student’s *t*-tests were used for statistical comparisons. Differences between groups were considered significant at *p* < 0.05.

## Results

### Properties of Rebound Depolarization

Membrane potential recordings were obtained from layer V mPFC pyramidal neurons in slices isolated from adult rats (58–62 days old). Glutamatergic and GABAergic transmission blockers were routinely added to the extracellular solution. The extracellular solution also contained TTX (0.5 μM, except for the results presented in Figures 2A–C). Therefore, the tested pyramidal neurons were synaptically isolated. The input resistance and cell membrane capacitance of tested pyramidal neurons were 110.7 ± 4.6 MΩ (*n* = 181) and 224.5 ± 29.8 pF (*n* = 192), respectively.

**Figure 1:**
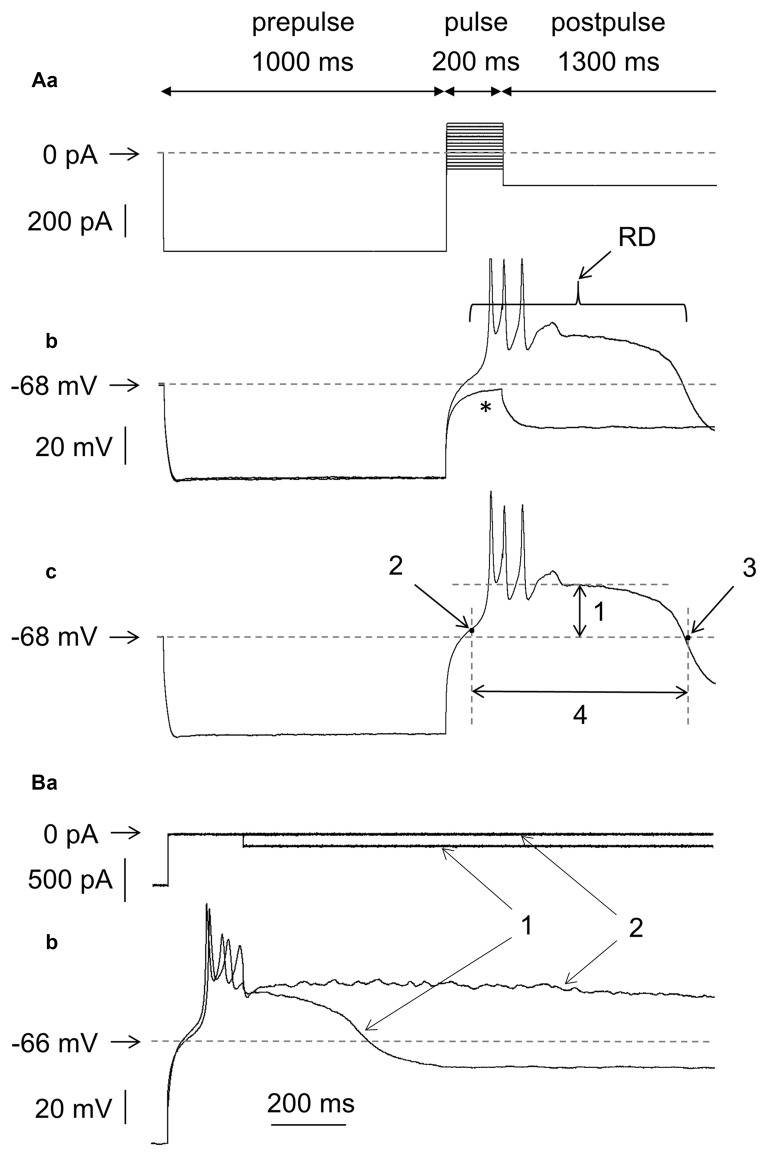
Measurements of the amplitude and duration of rebound depolarization (RD) in medial prefrontal cortex (mPFC) pyramidal neurons. **(Aa)** Standard protocol applied to study RD. A 600-pA hyperpolarizing current prepulse lasting 1000 ms preceded 200-ms current pulses applied in 20-pA increments from −100 pA to +180 pA. The 200-ms current pulses were followed by a hyperpolarizing current postpulse at −200 pA that lasted 1300 ms. **(b)** Membrane potential changes evoked by current pulses −20 pA (*) and 0 pA (RD) in the bath in the absence of Ca^++^. **(c)** An RD taken from **(b)**. Double-headed arrow 1 indicates maximum depolarization above the resting membrane potential attained after repetitive tetrodotoxin (TTX)-resistant spikelets. Arrow 2 indicates the beginning of RD when the depolarization phase of the RD attained 10% of its maximum level. Arrow 3 indicates the RD end, which was defined as the time point at which the RD repolarization phase intersected with the resting membrane potential level. Double-headed arrow 4 indicates the RD duration, defined as the time interval between the beginning and the end of the RD. **(B)** Voltage changes **(b1,b2)** evoked by a current pulse to 0 pA **(a)** followed by postpulses to −200 pA **(a1)** and postpulse 0 pA **(a2)**. The dotted horizontal line in panel **(Aa)** indicates 0 pA and the dotted horizontal lines in panel **(Ab,c,Bb)** and other figures indicate the resting membrane potential level.

**Figure 2:**
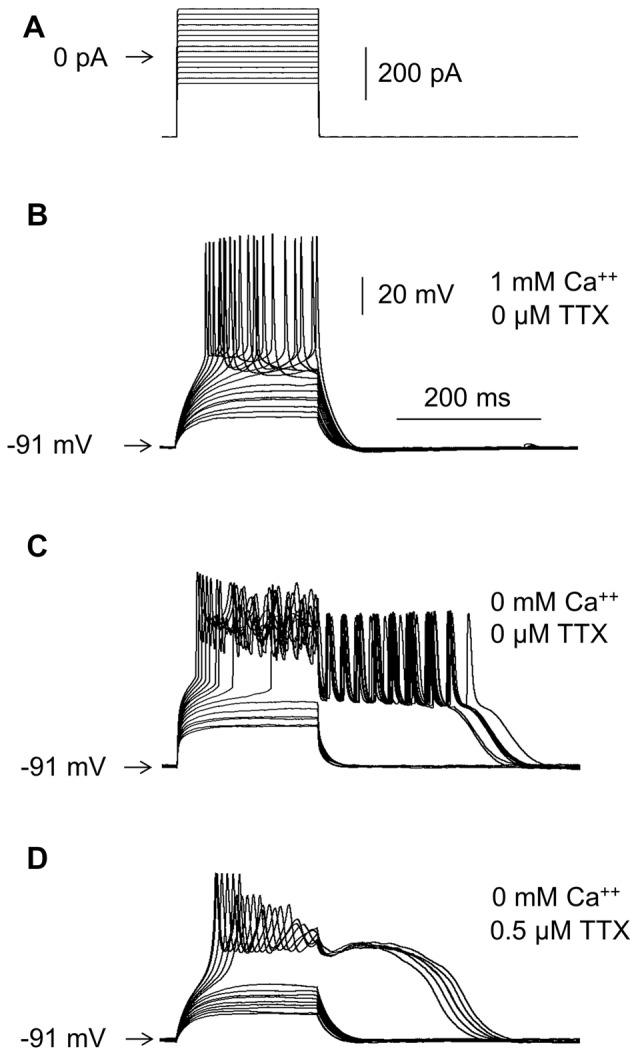
Effects of Ca^++^ and TTX in the extracellular solution on RD. **(A)** Portion of the current protocol applied to evoke RD shown in **(B–D)**. Current pulses were preceded by a −300-pA hyperpolarizing current prepulse lasting 1000 ms, followed by a −300-pA current postpulse lasting 1300 ms. Membrane potential changes evoked in the presence of 1 mM of Ca^++^ and the absence of TTX **(B)**, in the absence of Ca^++^ and the absence of TTX **(C)**, and in the absence of Ca^++^ and the presence of TTX (0.5 μM) **(D)** in the extracellular solution.

RD was typically evoked in the neurons as follows (exceptions are indicated in the text): the membrane potential was hyperpolarized using a negative current step of −600 pA that lasted 1000 ms (Figure 1Aa, prepulse). The hyperpolarizing prepulse was followed by current pulses lasting 200 ms each and applied in 20-pA increments from the −100-pA negative to +180-pA positive current steps (with respect to the “0” current level, Figure 1Aa). These current pulses were followed by a negative current postpulse of −200 pA that lasted 1300 ms (Figure 1Aa, postpulse). In Figure 1Ab are shown membrane potential changes evoked by only two (from 15 shown in Figure 1Aa) current pulses: to −20 pA and to 0 pA, lasting 200 ms each. The −20-pA current pulse was subthreshold to evoke RD (Figure 1Ab**). Current pulsed to 0 pA evoked RD that markedly outlasted the 200-ms current pulse duration (Figure 1Ab, RD). In the next Figures, usually two current pulses lasting 200 ms are shown: one just subthreshold and one threshold for RD, together with the subthreshold and threshold voltage responses (even though the voltage responses to all 200-ms current pulses shown in Figure 1Aa have been recorded). If RD could not be evoked, more than two 200-ms depolarizing current pulses together with voltage responses evoked by these current pulses were shown to demonstrate that high-threshold RDs were absent.

An additional hyperpolarizing current postpulse lasting 1300 ms (−200 pA, postpulse in Figure 1Aa) after 200-ms “pulses” was applied to shorten the RD duration. Without this additional postpulse, the RD duration was frequently longer than arbitrarily chosen by our analysis time (Figures 1Ba2,b2). Addition of a hyperpolarizing postpulse shortened the RD duration (Figures 1Ba1,b1).

The average 200-ms maximum current pulse that was subthreshold to evoke RD was −9.3 ± 3.8 pA (*n* = 192, negative current pulse with respect to the “0” current level). The average minimum (threshold) current pulse that invariably evoked RD was +10.7 ± 3.8 pA (*n* = 192, positive current with respect to the “0” current level). Therefore, the RD threshold corresponded to the current pulse amplitude in-between −9 pA and +11 pA—a value close to the 0-pA current level (a level that did not evoke a change in the resting membrane potential).

The amplitude of the RD was expressed as maximum depolarization above the resting membrane potential measured after the spikelets (after repetitive TTX-resistant fluctuating broad depolarizations seen at the beginning of the RD plateau, Figure 1Ac1). It was arbitrarily assumed that RD began when the depolarization phase of the RD attained 10% of its maximum level (Figure 1Ac2). The end of the RD was defined as the time point at which the RD repolarization phase intersected with the resting membrane potential level (Figure 1Ac3). The time interval between the beginning and the end of the RD was defined as the RD duration (Figure 1Ac4). RDs were evoked in 91% (192 out of 211) of the tested layer V pyramidal neurons. The resting membrane potential in these neurons was −67.9 ± 0.95 mV (*n* = 192), the amplitude of the RD was 30.6 ± 1.2 mV (*n* = 192), and the RD duration was 564.3 ± 39.6 ms (*n* = 192). These measurements were obtained for RDs evoked by the threshold current pulse.

RD was never evoked in the presence of Ca^++^ in the extracellular solution (tested at Ca^++^ concentrations of 0.1 mM, 0.3 mM and 1 mM, as shown in Figure 2B), unless the Ca^++^ (Figures 10Ac,d,Bc–e) or BK (Figures 7Ac,Ba) channel current was blocked, Ca^++^ was chelated in the intracellular solution (Figures 8Bb,Cb), or protein kinase C (PKC) was activated (Figures 11Ac,e). RD was also evoked in the absence (Figure 2C) and presence of 0.5, 1 and 10 μM of TTX in the extracellular solution (Figure 2D, shown in the presence of 0.5 μM of TTX). Moreover, in the absence of TTX, irrespective of the presence (Figure 2B) or absence (Figure 2C) of Ca^++^ in the bath, typical large-amplitude action potentials were evoked during depolarization. Small-amplitude, broad, repetitive depolarizations (spikelets) were evoked in the absence of Ca^++^, despite the presence of TTX in the bath (for example, Figure 2D).

**Figure 3:**
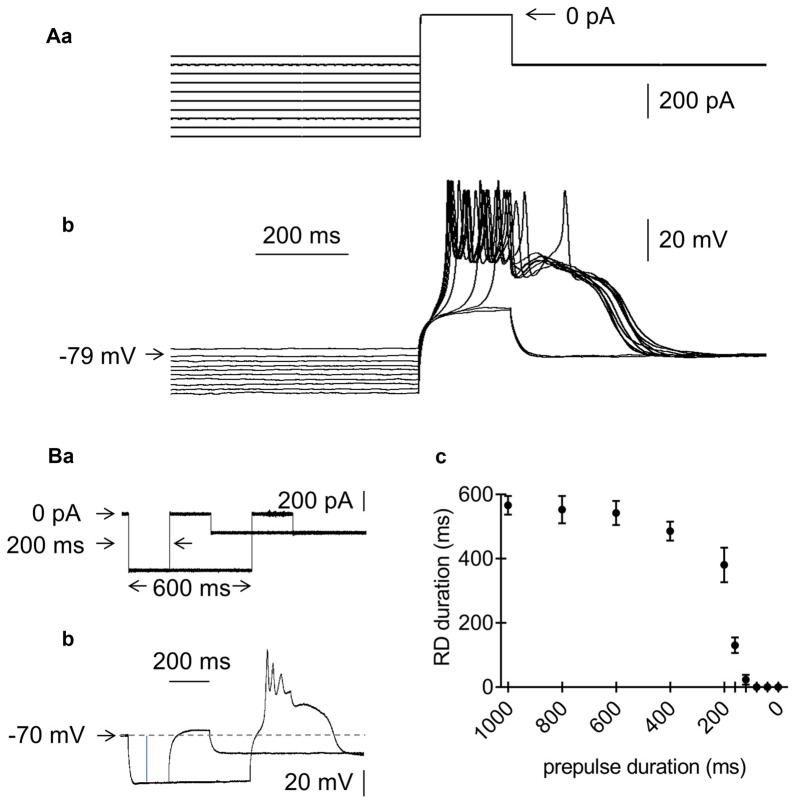
Effects of the amplitude and duration of the prepulse on RD in the absence of Ca^++^ in the extracellular solution. **(Aa)** Current protocol applied to study the effect of the prepulse amplitude on RD. Hyperpolarizing 1000-ms current prepulses from −700 pA to −250 pA were applied in 50-pA increments preceding the threshold current pulse to 0 pA, lasting 200 ms. Current pulses that evoked RD were followed by hyperpolarization to −300 pA, lasting 1300 ms. **(b)** RDs evoked by the current protocol shown in **(a)**. RDs were evoked only at prepulse potentials more negative than −79 mV. **(Ba)** Current protocol applied to study the effect of the prepulse duration on the RD duration. The prepulse amplitude was −600 pA. The prepulse duration was changed from 1000 to 0 ms. Only 2 prepulses with durations of 200 and 600 ms are shown. The 200-ms threshold current pulse that evoked RD was 0 pA. The postpulse amplitude was −200 pA and lasted 1300 ms. **(b)** RD was evoked at a prepulse duration of 600 ms, but not at a prepulse duration of 200 ms (protocol of current pulses is shown in **a**). **(c)** Relationship between prepulse duration (horizontal axis) and RD duration (vertical axis).

**Figure 4:**
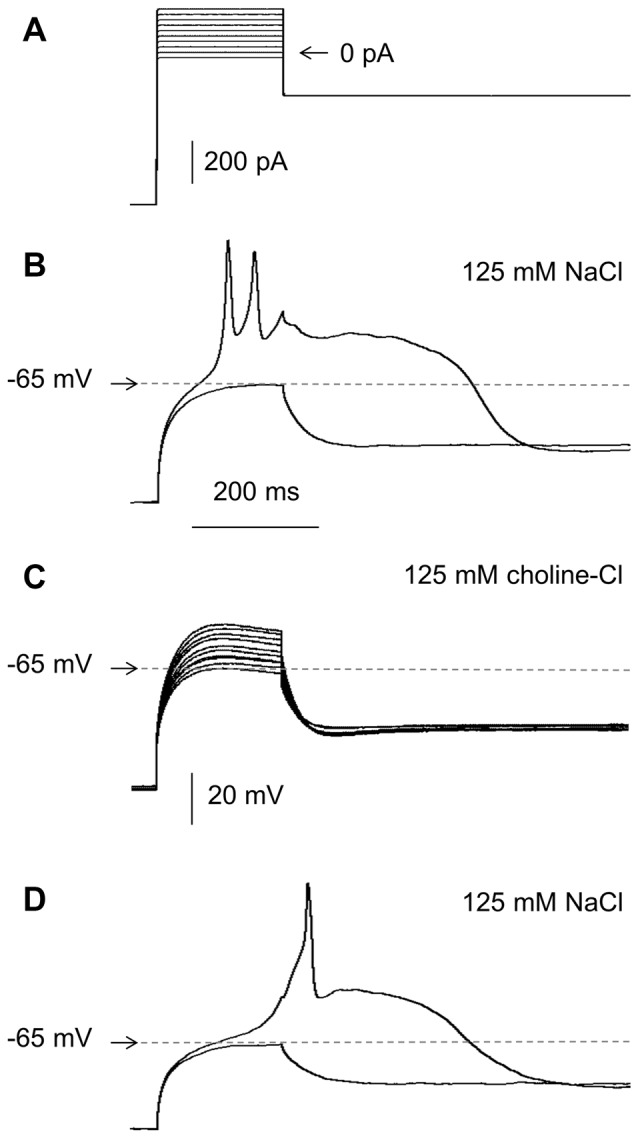
Effects of the extracellular concentration of Na^+^ on RD in the absence of Ca^++^ in the extracellular solution. **(A)** Portion of the current protocol applied to evoke the membrane potential changes shown in **(B–D)** (compare to Figure 1Aa). **(B)** RD evoked in the presence of 125 mM of NaCl in the extracellular solution. **(C)** Membrane potential changes recorded after replacing 125 mM of NaCl with 125 mM of choline-Cl in the extracellular solution. **(D)** RD evoked after choline chloride washout (replacing 125 mM of choline chloride with 125 mM of NaCl).

**Figure 5:**
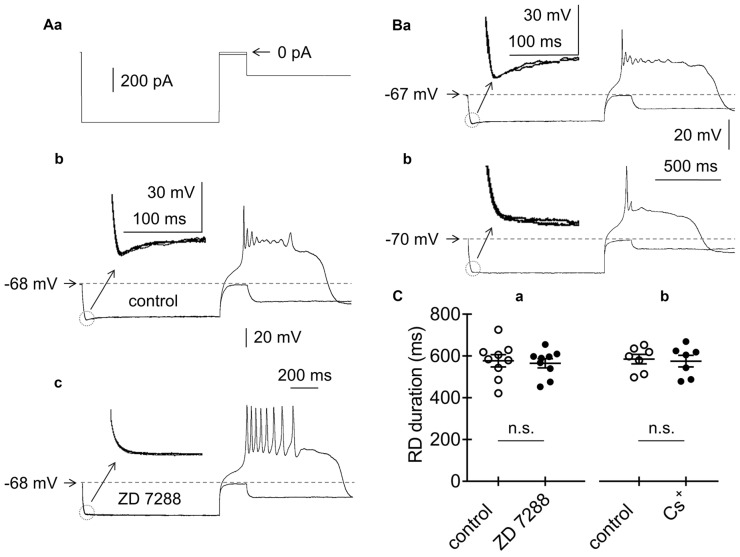
Effects of HCN channel blockers on RD in the absence of Ca^++^ in the extracellular solution. **(Aa)** Current protocol applied to evoke the membrane potential changes shown in **(b,c)** (compare to Figure 1Aa). RD evoked before **(b)** and during **(c)** bath application of ZD 7288 (50 μM). Insets to **(b,c)** show amplified voltage responses to the beginning of the rectangular hyperpolarizing current steps. Voltage sag was present before (inset to **b**) and was absent during bath application of ZD 7288 (50 μM, inset to **c**). **(B)** Examples of RDs evoked in two pyramidal neurons, in which voltage sag was present **(a)** and absent **(b)**. Insets show amplified voltage responses to the beginning of the rectangular hyperpolarizing current steps. The current protocol applied to evoke RD is shown in Figure 1Aa. **(C)** Duration of RD before (control) and after 15-min bath application of either ZD 7288 (**a**, ZD 7288, 50 μM) or Cs^+^ (**b**, Cs^+^, 3 mM); n.s., non-significant.

**Figure 6:**
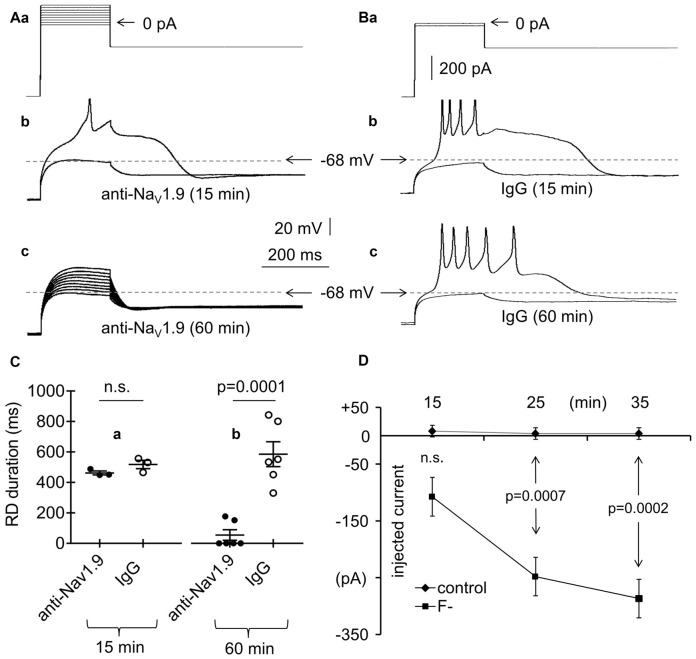
Effects of an anti-Nav1.9 channel antibody, IgG and F^−^ on RD in the absence of Ca^++^ in the extracellular solution. **(Aa)** Current protocol applied to evoke RD (compare Figure 1Aa). **(b)** Voltage responses to subthreshold −20-pA and threshold 0-pA current pulses lasting 200 ms are shown. Voltage responses were recorded after the 15-min pipette presence of anti-Nav1.9 channel antibody (4 μg/ml, anti-Nav1.9, 15 min). **(c)** Voltage responses to currents steps shown in **(a)** after loading the cell for 60 min with anti-Nav1.9 channel antibody (4 μg/ml, anti-Nav1.9, 60 min). In **(b,c)** are shown recordings obtained from the same neuron. **(B)** Current protocol applied to evoke RD shown in **(b,c)**.** (a)** Voltage response to −20 pA subthreshold and 0 pA threshold current pulses lasting 200 ms recorded after the 15 **(b)** and 60 min **(c)** pipette presence of IgG (4 μg/ml; IgG, 15 min and IgG, 60 min, respectively). **(C)** Mean RD durations after 15-min **(a)** and 60-min **(b)** intracellular applications of anti-Nav1.9 antibody (anti-Nav1.9) or IgG (IgG). **(D)** RD current thresholds recorded during 15, 25 and 35 min in the presence of either only Cl^−^ (control) or Cl^−^ and F^−^ (F^−^) in the pipette solution; n.s., non-significant.

**Figure 7:**
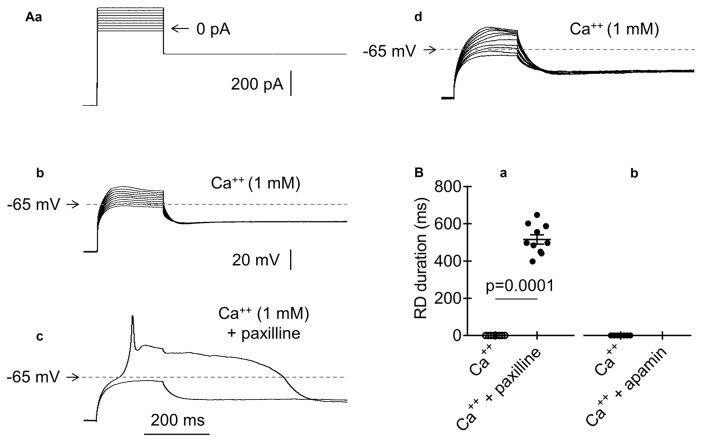
Effects of BK and SK channel blockers on RD in the presence of Ca^++^ (1 mM) in the extracellular solution. **(Aa)** The current protocol (compare to Figure 1Aa) applied to evoke membrane potential changes shown in **(b–d)**. Membrane potential changes evoked before **(b)**, after a 15-min application of paxilline (10 μM) **(c)**, and after paxilline washout **(d)**. **(c)** Membrane potential changes to 200-ms pulses to −40 pA (subthreshold step) and to −20 pA (threshold step). **(B)** Absence of RD in the presence of only Ca^++^ (1 mM) in the extracellular solution (**Ba,b**, Ca^++^). Duration of RD and the presence of Ca^++^ (1 mM) together with paxilline (**a**, 10 μM, Ca^++^ + paxilline). Absence of RD in the bath presence of Ca^++^ (1 mM) together with apamin (**b**, 50 nM, Ca^++^ + apamin).

**Figure 8:**
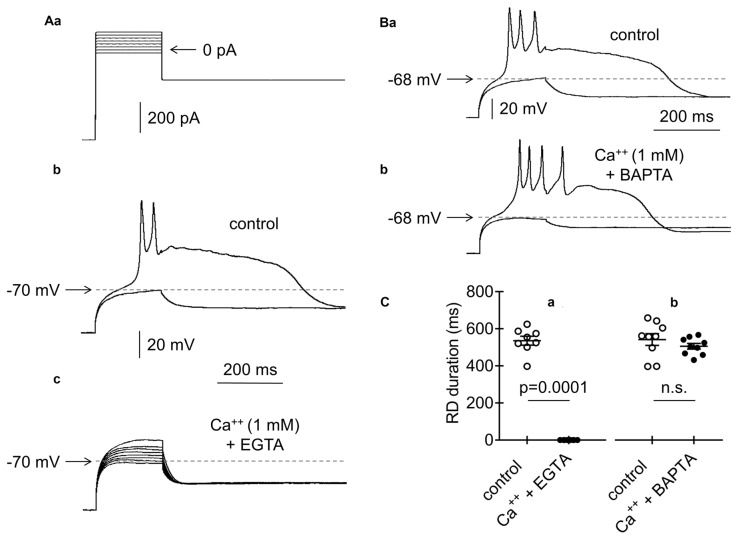
Effects of intracellular application of EGTA or BAPTA on RD. **(Aa)** Current protocol applied to evoke RDs shown in **(Ab,c,Ba,b)** (compare Figure 1Aa). **(b)** Voltage responses evoked by pulse amplitudes of 0 pA (subthreshold) and +20 pA (threshold required to evoke RD). RD was evoked at the beginning of cell “dialysis” with EGTA (10 mM) in the absence of Ca^++^ in the extracellular solution (control). **(c)** RD was not evoked after 60 min of cell “dialysis” with EGTA in the bath presence of Ca^++^ (1 mM, Ca^++^ + EGTA) (**b** and **c** show the results obtained from the same neuron). **(Ba)** RD was evoked at the beginning of cell “dialysis” with BAPTA (100 μM) in the absence of Ca^++^ (control). **(b)** RD was evoked in the same neuron after 60 min of cell “dialysis” with BAPTA despite the presence of Ca^++^ in the extracellular solution (1 mM, Ca^++^ + BAPTA). Voltage responses were evoked by 200-ms current steps to 0 pA (subthreshold) and to +20 pA (threshold current step required to evoke RD). **(C)** The RD duration measured at the beginning of cell “dialysis” with EGTA (**a**, control) or BAPTA (**b**, control) in the absence of Ca^++^ in the extracellular solution. RD could not be evoked after 60 min of cell dialysis with EGTA (**a**, Ca^++^ + EGTA) in the bath presence of Ca^++^ (1 mM). RD was evoked after 60 min of cell dialysis with BAPTA (**b**, Ca^++^ + BAPTA), despite the presence of Ca^++^ (1 mM) in the extracellular solution; n.s., non-significant.

**Figure 9:**
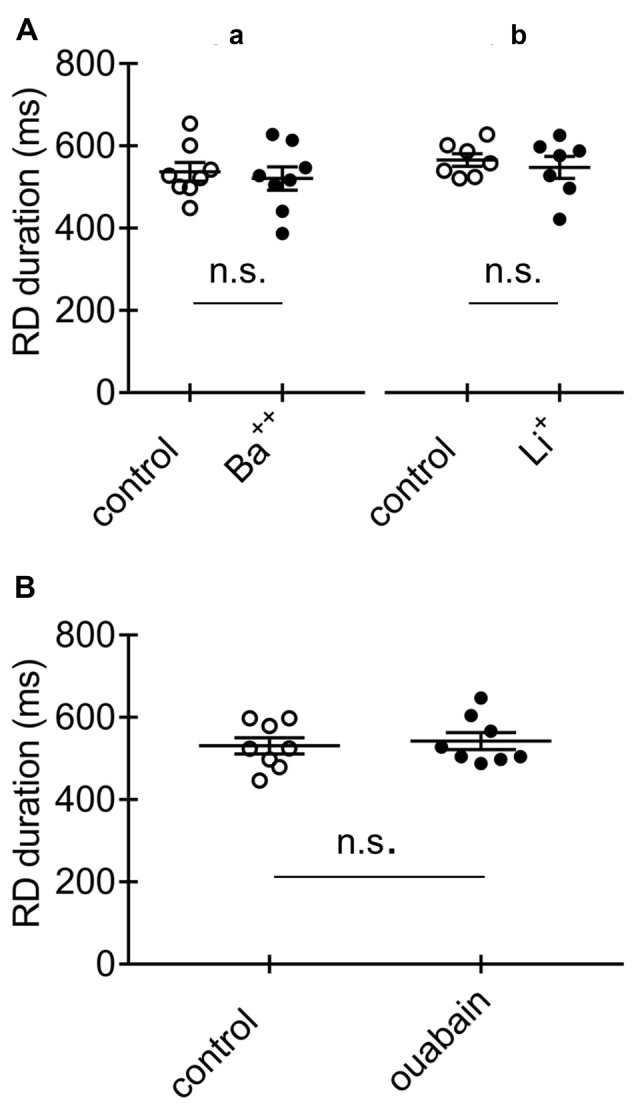
Effects of Ba^++^, Li^+^ and ouabain on RD in the absence of Ca^++^ in the extracellular solution. **(A)** Duration of RD before (control) and after a 15-min application of Ba^++^ (Ba^++^, 200 μM) to the extracellular solution **(a)** and before (control) and after replacing 125 mM NaCl with 125 mM LiCl (Li^+^) in the extracellular solution **(b)**. **(B)** Duration of RD before (control) and after a 15-min application of 100 μM of ouabain (ouabain). The threshold RDs were evoked by application of a standard protocol shown in Figure 1Aa.

**Figure 10:**
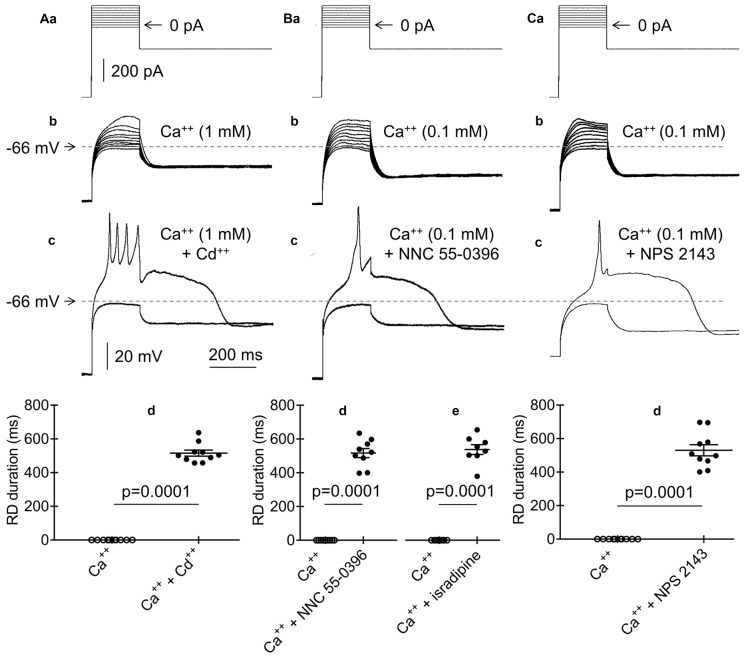
Effects of Cd^++^, NNC 55-0396, isradipine and NPS 2143 on RD. **(Aa)** Current protocol applied to evoke voltage changes shown in **(b,c)** (compare Figure 1Aa). **(b)** RD was not evoked in the presence of Ca^++^ (1 mM) and the absence of Cd^++^ in the bath in response to 200-ms current pulses from −20 pA to +160 pA. **(c)** RD evoked by a threshold 200-ms current pulse to 0 pA in the bath presence of Ca^++^ (1 mM) and Cd^++^ (50 μM). **(d)** Absence of the RD in the bath in the presence of only Ca^++^ (Ca^++^, 1 mM). RD could be evoked in the bath in the presence of Ca^++^ (1 mM) together with Cd^++^ (50 μM, Ca^++^ + Cd^++^). **(Ba)** Current protocol applied to evoke voltage changes shown in **(b,c)** (compare Figure 1Aa). **(b)** RD was not evoked in the presence of Ca^++^ (0.1 mM) in the bath in response to 200-ms current pulses from −20 pA to +160 pA. **(c)** RD evoked by a threshold 200-ms current pulse to 0 pA in the presence of Ca^++^ (0.1 mM) together with the T-type Ca^++^ channel blocker NNC 55-0396 (50 μM) in the bath. **(d)** The RD duration in the presence of only 0.1 mM of Ca^++^ (Ca^++^) or Ca^++^ (0.1 mM) together with the T-type channel blocker NNC 55-0396 (50 μM) (**d**, Ca^++^ + NNC 55-0396) in the extracellular solution. **(e)** The RD duration in the presence of only Ca^++^ (Ca^++^, 0.1 mM) or Ca^++^ (0.1 mM) together with the L-type channel blocker isradipine (10 μM; Ca^++^ + isradipine) in the extracellular solution. **(Ca)** Current protocol applied to evoke voltage changes shown in **(b,c)** (compare Figure 1Aa). **(b)** RD was not evoked in the presence of only Ca^++^ (0.1 mM) in the bath in response to 200-ms current pulses from −20 pA to +160 pA. **(c)** RD was evoked in response to a 200-ms threshold current pulse to 0 pA in the presence of Ca^++^ (0.1 mM) together with calcium sensing receptor (CaSR) blocker (NPS 2143, 3 μM) in the bath. **(d)** The RD was not evoked in the presence of only 0.1 mM of Ca^++^ (Ca^++^). RD duration in the bath presence Ca^++^ (0.1 mM) together with NPS 2143 (3 μM) (Ca^++^ + NPS 2143).

**Figure 11:**
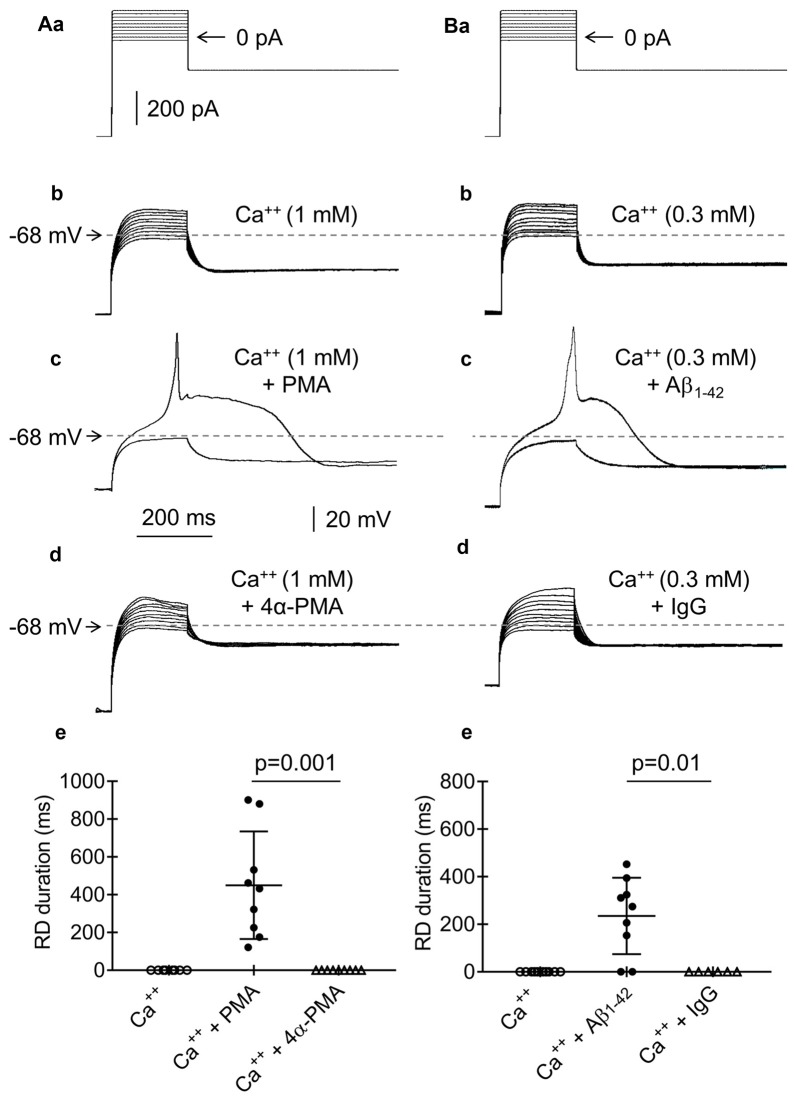
Effects of phorbol 12-myristate 13-acetate (PMA), 4αPMA, Aβ_1–42_ and IgG on RD. **(Aa)** Current protocol applied to evoke voltage changes shown in **(b–d)** (compare Figure 1Aa). **(b)** RD was not evoked in response to 200-ms current pulses from −20 pA to +160 pA in the presence of Ca^++^ (1 mM) in the bath. **(c)** RD was evoked by the 200-ms threshold current pulse to 0 pA in the bath presence of PMA (1 μM) and Ca^++^ (1 mM). **(d)** The absence of RD in the presence of an inactive analog of PMA (4α-phorbol 12-myristate 13-acetate, 4α-PMA, 2 μM) together with Ca^++^ (1 mM) in the bath. **(e)** Absence of RD in the bath presence of only 1 mM of Ca^++^ (Ca^++^) or Ca^++^ (1 mM), together with an inactive analog of the protein kinase C (PKC) activator 4α-PMA (2 μM, Ca^++^ + 4α-PMA). Duration of RD in the bath presence of Ca^++^ (1 mM) together with the PKC activator PMA (1 μM, Ca^++^ + PMA). The voltage traces shown in **(b,c)** were obtained from the same neuron. **(Ba)** Current protocol applied to evoke voltage changes shown in **(b–d)** (compare Figure 1Aa). **(b)** RD was not evoked by current pulses from −20 pA to +160 pA in the presence of Ca^++^ (0.3 mM) in the bath. **(c)** RD was evoked by a threshold 0 pA 200-ms pulse when the pipette solution contained Aβ_1–42_ (10 μM) and Ca^++^ (0.3 mM) was present in the bath. **(d)** Absence of RD when the pipette solution contained IgG (4 μg/ml) and the extracellular solution contained Ca^++^ (0.3 mM). The voltage traces shown in **(b,c)** were obtained from the same neuron. **(e)** The absence of RD in the bath presence of Ca^++^ (0.3 mM) only or in the bath presence of Ca^++^ (0.3 mM) together with IgG (4 μg/ml, Ca^++^ + IgG) in the pipette solution. The RD was evoked in 7 from 9 tested neurons in the bath presence Ca^++^ (0.3 mM), together with the presence of Aβ_1–42_ (10 μM) in the pipette solution (Ca^++^ + Aβ_1–42_).

The effects of the hyperpolarizing prepulse amplitude and duration were also examined during the absence of Ca^++^ in the bath. To examine the effect of the prepulse level on RD, we applied 1000-ms hyperpolarizing current prepulses from −700 pA to −250 pA in 50-pA increments before the 200-ms threshold current pulse that evoked RD (Figure 3Aa). The postpulse amplitude was −300 pA. RD was abolished in an all-or-none fashion at prepulse membrane potential levels above −81.5 ± 3.1 mV (*n* = 10, Figure 3Ab).

The hyperpolarizing prepulse duration on RD was tested as follows. At a prepulse amplitude of −600 pA, the prepulse durations were as follows (in ms): 1000, 800, 600, 400, 200, 160, 120, 80, 40 and 0. Figures 3Ba,b shows RD evoked with a prepulse duration of 600 ms and the lack of RD with a prepulse duration of 200 ms. With a prepulse duration shorter than 600 ms, the RD duration also became gradually shorter, and RD was abolished with a prepulse duration shorter than 148.6 ± 47.4 ms (*n* = 7, Figure 3Bc).

The above results indicated that the effector responsible for RD formation in mPFC pyramidal neurons had the following properties: (a) it was evoked in the absence of Ca^++^ in the extracellular solution, (b) it was TTX-resistant, and (c) it was activated after the hyperpolarizing current step below −81 mV and lasting longer than 150 ms. Its threshold after the hyperpolarizing step was close to the resting membrane potential level.

### Effector Responsible for RD

Because RD was blocked in the presence of Ca^++^ in the extracellular solution, the search for RD effectors was performed in the absence of Ca^++^ in the bath.

The inward Na^+^ current was responsible for RD because it was completely abolished when 125 mM of NaCl was replaced with 125 mM of choline-Cl in the extracellular solution and was recovered when the Na^+^ gradient was restored (*n* = 7, Figures 4A–D).

A number of membrane proteins may be responsible for Na^+^-dependent RD. HCN channels, which are permeable to Na^+^ and K^+^ and are inactivated in the steady-state at the resting membrane potential, are possible candidate channels, as suggested by others (Albertson et al., 2011). Layer V pyramidal neurons that project to subcortical structures have been shown to express this current, while those that project to the cortex do not (Dembrow et al., 2010; Lee et al., 2014). The presence of HCN currents is marked by a voltage sag at the beginning of the hyperpolarizing voltage step (Dembrow et al., 2010; Albertson et al., 2011; Lee et al., 2014). Indeed, in our study, bath application of a specific blocker of HCN channels (ZD 7288, 50 μM, *n* = 5) abolished the voltage sag seen at the beginning of prepulse hyperpolarization (compare insets to Figures 5Ab,c). Moreover, RD depolarization in the tested pyramidal neurons was found in cells with (Figure 5Ba and inset) and without (Figure 5Bb and inset) a voltage sag. Furthermore, application of the HCN channel blockers ZD 7288 (50 μM) or Cs^+^ (3 mM) to the extracellular solution did not affect the duration of RD. After a 15-min bath application of ZD 7288 (ZD 7288; 533.9 ± 18.6 ms, *n* = 9, Figures 5Aa,c) or Cs^+^ (555.6 ± 27.6 ms, *n* = 7, traces not shown), the duration of RD did not significantly differ from that of the RD duration measured immediately before blocker application (557.1 ± 29.1 ms, *n* = 9, *p* > 0.05, Figure 5Ca and 565.2 ± 22 ms, *n* = 7, *p* > 0.05, Figure 5Cb, respectively). This finding indicated that HCN channels were not responsible for RD in mPFC pyramidal neurons.

We also considered whether NALCN (Na^+^-leak channel) currents, which are inhibited in the presence of Ca^++^ in the extracellular solution and are preferentially expressed in neurons (Cochet-Bissuel et al., 2014), might be responsible for RD. A 15-min bath application of NALCN blockers, including Gd^+++^ (100 μM) or verapamil (1 mM; Boone et al., 2014), did not prevent RD. The RD durations following Gd^+++^ (580.2 ± 14.7 ms, *n* = 7) or verapamil application (540.6 ± 33.5 ms, *n* = 8) did not differ significantly from those measured before blocker application (571.2 ± 12.1 ms, *n* = 7, *p* > 0.05 and 558.9 ± 28.8 ms, *n* = 8, *p* > 0.05, respectively). The effects of Gd^+++^ and verapamil on RD are not shown in the figures.

Persistent (i.e., non-inactivating over time) Na^+^ currents have also been proposed to be responsible for RD (Sangrey and Jaeger, 2010). It was recently suggested that low-threshold and TTX-resistant Nav1.9 Na^+^ channel currents are present in layer V mPFC pyramidal neurons (Kurowski et al., 2015; Gawlak et al., 2017). To test whether these channels are responsible for RD, an antibody against Nav1.9 channels (4 μg/ml) was applied through the recording pipette to the tested pyramidal neurons. The RD durations were 462.5 ± 12.6 ms (*n* = 3; Figures 6Ab,Ca, anti-Nav1.9, 15 min) and 54.8 ± 34.8 ms (*n* = 6; Figures 6Aa,c,Cb, anti-Nav1.9, 60 min) when measured at 15 and 60 min, respectively, after application of the antibody. In four out of six cases, after 60 min of intracellular presence of anti-Nav1.9 antibody, the RD was completely abolished (Figures 6Aa,c,Cb). As a control, normal IgG (4 μg/ml) was applied to the cells. The RD durations measured at 15 and 60 min after the onset of cell “dialysis” with IgG were 517.8 ± 27.3 ms (*n* = 3; Figures 6Ba,b,Ca, IgG, 15 min) and 585.3 ± 115.3 ms (*n* = 6; Figures 6Ba,c,Cb, IgG, 60 min). The RD durations measured during the intracellular application of IgG were not significantly different (unpaired Student’s *t*-test, *p* > 0.05) from those measured in the absence of any tested biologically active compounds in the extra- and intracellular solutions (564.3 ± 39.6 ms, *n* = 192). The duration of RD was significantly shorter in the presence of the anti-Nav1.9 antibody than that in the presence of IgG (*p* = 0.0001, unpaired Student’s *t*-test) when measured at 60 min after antibody application to the cells (Figure 6Cb).

Others (Coste et al., 2004) have indicated that in the presence of F^−^ in the pipette solution, the threshold of the inward Nav1.9 currents shift toward hyperpolarization. Therefore, we measured the current threshold of RD when only Cl^−^ anions were present and when 20 mM of KCl was replaced with 20 mM of KF in the pipette solution. The threshold was expressed as minimum amplitude of the 200-ms current pulse applied after the hyperpolarizing prepulse (Figure 1Aa) that evoked RD. The RD thresholds were +8.0 ± 10.2 pA (*n* = 5), +4.0 ± 16.0 pA (*n* = 5), and +4.0 ± 14.7 pA (*n* = 5) measured at 15, 25 and 35 min after the onset of cell “dialysis” with standard Cl^−^-containing solution (Figure 6D, control). The RD thresholds were −107.5 ± 44.1 pA (*n* = 8), −248.0 ± 45.0 pA (*n* = 5), and −286.7 ± 43.7 pA (*n* = 3) at 15, 25, and 35 min after the onset of cell “dialysis” with F^−^ (Figure 6D, F^−^). The RD threshold was significantly decreased after 25 min (*p* = 0.0007, unpaired Student’s *t*-test) and 35 min (*p* = 0.0002, unpaired Student’s *t-test)* of cell “dialysis” with F^−^ compared with the RD thresholds measured at the same time points in the presence of only Cl^−^ anions in the intracellular solution (Figure 6D).

These results suggest that the low-threshold and TTX-resistant inward Na^+^ current, presumably through Nav1.9 channels, is responsible for RD in pyramidal neurons.

### Effector Responsible for Restraining RD

RDs were not evoked in the presence of Ca^++^ in the extracellular solution. It has been thoroughly documented that the removal of Ca^++^ from the extracellular solution decreases the baseline Ca^++^ concentration in the cytoplasm in both neurons (Magee et al., 1996; Pinilla et al., 2005; Cheek and Thorn, 2006; Nichols et al., 2007) and nonneuronal cells (Mignen et al., 2017). Decreasing the Ca^++^ concentrations in the cytoplasm leads to a markedly increased voltage threshold of BK channels (large conductance Ca^++^-activated K^+^ channels; Berkefeld and Fakler, 2013). We assumed that the depletion of Ca^++^ in the extracellular solution lowers the Ca^++^ levels in the cytoplasm and inhibits the outward K^+^ current through BK channels, allowing for the emergence of Na^+^-dependent RD. To test this hypothesis, a standard current-clamp protocol was applied (Figure 7Aa). As expected, RD was not evoked in the presence of Ca^++^ (1 mM) in the extracellular solution (Figures 7Ab,Ba, Ca^++^). When a BK channel blocker (paxilline, 10 μM; Książek et al., 2013) was applied to the bath, a typical RD was elicited, despite the presence of Ca^++^ (1 mM) in the extracellular solution (Figures 7Ac,Ba, Ca^++^ + paxilline). RD was eliminated after paxilline washout (Figure 7Ad). The duration of RD in the presence of Ca^++^ and paxilline was 516.6 ± 25.1 ms (*n* = 10) and did not differ from the RD duration measured in the absence of Ca^++^ or any other tested compound in the extracellular solution (564.3 ± 39.6 ms, *n* = 192, *p* > 0.05, unpaired Student’s *t*-test). The resting membrane potentials before (−67.5 ± 1.2 mV) and after (−67.9 ± 0.8 mV) 15-min bath application of paxilline (10 μM) were not significantly different (*n* = 10, *p* > 0.05).

Ca^++^-dependent SK-type K^+^ channels are also expressed in mPFC pyramidal neurons (Faber, 2010). Therefore, we also examined the putative involvement of SK currents in RD. When Ca^++^ (1 mM) was present in the extracellular solution, RD could not be evoked, irrespective of the absence (Figure 7Bb, Ca^++^, *n* = 7) or 15-min presence of a selective SK channel blocker (apamin, 50 nM; Figure 7Bb, Ca^++^ + apamin, *n* = 7) in the bath. This finding suggests that SK channels are not involved in the modulation of RD.

Chelators of Ca^++^ (EGTA, 10 mM or BAPTA, 100 μM) were applied to the pipette solution to further evaluate the involvement of BK channels in the abolishment of RD. RD was evoked using a standard protocol (Figure 8Aa, compare Figure 1Aa). Immediately after gaining access to the cytoplasm with the recording pipette containing EGTA, typical RD was evoked in the absence of Ca^++^ in the extracellular solution (Figures 8Ab,Ca, control; 535.7 ± 24.2 ms, *n* = 8). In the same neurons, RD was not evoked 60 min after cell “dialysis” with EGTA in the presence of Ca^++^ (1 mM) in the bath (Figures 8Ac,Ca, Ca^++^ + EGTA). At the beginning of cell “dialysis” with BAPTA in the absence of Ca^++^ in the bath, the RD duration was 541.8 ± 31.7 ms (Figures 8Ba,Cb, control, *n* = 9). At 60 min after cell “dialysis” with BAPTA, despite the extracellular presence of Ca^++^ (1 mM), RD was evoked. Under these conditions, the RD duration was 505.6 ± 15.5 ms (Figures 8Bb,Cb, Ca^++^ + BAPTA, *n* = 9) and was not different from the RD duration measured at the beginning of cell “dialysis” with BAPTA (Figure 8Cb, *p* > 0.05). The obtained results indicated that intracellular Ca^++^ chelation with BAPTA abolished the inhibitory effect of BK channel activation on RD. The superior effectiveness of BAPTA over EGTA buffer in the elimination of the effect of Ca^++^ on BK channels has been well documented (Fakler and Adelman, 2008).

Cortical neurons also express Na^+^-dependent K^+^ currents (Slick and Slack channels, K_Na_; Uchino et al., 2003; Bhattacharjee et al., 2005). Na^+^ entering the cell during RD may activate K_Na_ currents and shorten the RD duration (Krey et al., 2010). Ba^++^ blocks K_Na_ channels (Bhattacharjee et al., 2005), while Li^+^, to which Na^+^ channels are permeable, has been shown to barely activate K_Na_ (Kaczmarek, 2013). Tests with Ba^++^ and Li^+^ were performed in the absence of Ca^++^ in the extracellular solution. We found that the duration of RD was 537.0 ± 22.6 ms (Figure 9Aa, control, *n* = 8) before and 520.8 ± 28.4 ms after 15-min bath application of Ba^++^ (200 μM; Figure 9Aa, Ba^++^, *n* = 8; *p* > 0.05). We examined whether replacement of 125 mM of NaCl with 125 mM of LiCl in the extracellular solution could influence the duration of RD. The durations of RD before (565.7 ± 15.5 ms; Figure 9Ab, control, *n* = 7) and after the replacement of Na^+^ with Li^+^ (547.7 ± 26.6 ms; Figure 9Ab, Li^+^, *n* = 7) were not significantly different (*p* > 0.05), suggesting that Na^+^-dependent K^+^ channels were not involved in shortening the RD duration.

Another consideration is that activation of Na^+^/K^+^-ATPase may participate in shortening the RD duration (Krey et al., 2010; Forrest et al., 2012). Na^+^/K^+^-ATPase may be activated by Na^+^ entering the cell during RD. Experiments were performed in the absence of Ca^++^ in the extracellular solution. A 15-min application of the Na^+^/K^+^-ATPase blocker ouabain (100 μM) did not prolong the RD evoked in pyramidal neurons. The durations of RD in the absence and presence of ouabain in the extracellular solution were 530.7 ± 19.9 ms (Figure 9B, control, *n* = 8) and 542.4 ± 20.5 ms (Figure 9B, ouabain, *n* = 8), respectively; these values were not significantly different (*p* > 0.05).

The above results suggest that RD is not detected in the bath in the presence of Ca^++^, most likely because Ca^++^ enters the cell cytoplasm and activates the outward K^+^ current through BK channels. The outward K^+^ current counterbalances the inward Na^+^ current and extinguishes RD.

### Route of Ca^++^ Entry Into the Cell Following the Hyperpolarization Current Step

The above results indicate that low-threshold Na^+^ and Ca^++^ channels are simultaneously activated when the membrane potential returns to its resting level after the hyperpolarization step.

To identify the route through which Ca^++^ passes from the extra- to the intracellular solution after the hyperpolarizing current step, we applied the nonselective Ca^++^ channel blocker Cd^++^ (50 μM) to the bath. The standard current-clamp protocol (compare Figure 10Aa) did not evoke RD in the presence of Ca^++^ (1 mM) and the absence of Cd^++^ (Figures 10Ab,d, Ca^++^, *n* = 10) in the bath. When Cd^++^ was added to the extracellular solution, RD depolarization was evoked, despite the extracellular presence of Ca^++^ (1 mM, Figure 10Ac). The RD duration under this condition was 515.9 ± 18.0 ms (Figure 10Ad, Ca^++^ + Cd^++^, *n* = 10) and did not differ from the RD duration recorded in the absence of Ca^++^ in the extracellular solution (*p* > 0.05, unpaired Student’s *t*-test, 564.3 ± 39.6 ms, *n* = 192).

Low-threshold T-type Ca^++^ channels are de-inactivated after membrane hyperpolarization (Molineux et al., 2006; Zamponi et al., 2010). To identify T-type channels as a potential route of Ca^++^ entry into the cytoplasm after the hyperpolarization current step, we applied the highly selective T-type channel blocker NNC 55-0396 (50 μM) to the bath. As expected, RD was not evoked in the presence of 0.1 mM of Ca^++^ and the absence of the T-type channel blocker (Figures 10Ba,b,d, Ca^++^, *n* = 9). However, RD was evoked in the presence of 0.1 mM of Ca^++^ and NNC 55-0396 (516.7 ± 27.0 ms; Figures 10Bc,d, Ca^++^ + NNC 55-0396, *n* = 9). At higher Ca^++^ concentrations (0.3 mM and above), RD could not be evoked, despite the presence of NNC 55-0396 in the bath.

A low-threshold voltage-dependent L-type Ca^++^ channel current has been described (Magee et al., 1996; Lipscombe et al., 2004; Navedo et al., 2005; Kolaj et al., 2016). Isradipine is an effective blocker of this current (Campbell et al., 1997; Anekonda et al., 2011). In our study, bath application of isradipine (10 μM) permitted RD (537.6 ± 29.0 ms, *n* = 8) in the presence of Ca^++^ in the bath at a concentration of 0.1 mM (Figure 10Be, Ca^++^ + isradipine). At higher Ca^++^ concentrations (0.3 mM and above), RD could not be evoked, despite the presence of this L-type channel blocker. In the presence of only Ca^++^ in the bath at a concentration of 0.1 mM, RD was not evoked (Figure 10Be, Ca^++^, original voltage tracings before and after isradipine are not shown).

Separate bath application of T- and L-type channel blockers eliminated the inhibitory effect of Ca^++^ on RD, provided that the Ca^++^ concentration in the extracellular solution did not exceed 0.1 mM. Therefore, we simultaneously applied T-type (NNC 55-0396, 50 μM) and L-type (isradipine, 10 μM) blockers to the bath and examined their effects on RD. RD could not be evoked using the standard current-clamp protocol (compare Figure 1Aa) in the presence of Ca^++^ (0.1 mM) and the absence of blockers in the bath. When 0.1 mM Ca^++^ was applied to the extracellular solution together with these two channel blockers, typical RD was evoked (561.3 ± 29.7 ms, *n* = 6). In the presence of Ca^++^ at concentrations of 0.3 mM and higher in the bath, RD could not be evoked, despite the presence of the T- and L-type channel blockers (original voltage tracings not shown in the figures).

Stimulation of the calcium sensing receptor (CaSR) by Ca^++^ has been found to activate BK channels (Vassilev et al., 1997; Chattopadhyay et al., 1999; Bandyopadhyay et al., 2007). Therefore, we tested whether inhibition of CaSR would lead to the inhibition of BK channels and enable RD. In the presence of 0.1 mM of Ca^++^ and the absence of a CaSR inhibitor, RD could not be evoked (Figures 10Ca,b,d, Ca^++^, *n* = 10). However, in the presence of a CaSR blocker (NPS 2143, 3 μM) and 0.1 mM of Ca^++^ in the bath, RD was evoked (530.4 ± 33.1 ms; Figures 10Cc,d, Ca^++^ + NPS 2143, *n* = 10). At a higher concentration of Ca^++^ (0.3 mM and above), RD could not be evoked, despite the presence of a CaSR blocker.

The above results indicate that Ca^++^ activating BK channels and attenuating Na^+^-dependent RD enters the cell through voltage-gated Ca^++^ channels, including T- and L-type Ca^++^ channels.

### Effects of PKC and Aβ_1–42_ on RD

Activation of PKC inhibits BK channels (Shipston and Armstrong, 1996; Schubert and Nelson, 2001; Tian and Laychock, 2001; Kizub et al., 2010; Zhou et al., 2010; van Welie and du Lac, 2011). Therefore, we tested the effects of a membrane-permeable PKC activator (PMA, 1 μM) applied to the extracellular solution on RD. In the absence of PMA and the presence of Ca^++^ in the extracellular solution (1 mM), RD could not be evoked (Figures 11Aa,b,e, Ca^++^, *n* = 9). However, in the same neurons, RD was evoked after a 15-min bath application of PMA, despite the extracellular presence of 1 mM of Ca^++^ (Figure 11Ac). The duration of RD evoked in the presence of PMA (449.9 ± 94.7 ms, Figure 11Ae, Ca^++^ + PMA, *n* = 9) did not differ from the duration of RD evoked in the absence of Ca^++^ in the extracellular solution (564.3 ± 39.6 ms, *n* = 192, *p* > 0.05, unpaired Student’s *t*-test). As a control for the effect of PMA, an inactive analog of PMA (4α-PMA, 2 μM) was applied to the bath. RD could not be evoked with a 15-min bath application of Ca^++^ (1 mM) and 4α-PMA (Figures 11Aa,d,e, Ca^++^ + 4α-PMA, *n* = 8, *p* = 0.001, unpaired Student’s *t*-test).

Aβ_1–42_ has been shown to inhibit BK channels (Yamamoto et al., 2011). To examine the effect of Aβ_1–42_ on RD, we added 10 μM of Aβ_1–42_ to the recording pipette solution. RD could not be evoked at the beginning of cell “dialysis” with Aβ_1–42_ and in the presence of Ca^++^ (0.3 mM; Figures 11Ba,b) in the bath. However, RD could be evoked after 60 min of cell “dialysis” with Aβ_1–42_, despite the presence of Ca^++^ in the extracellular solution (0.3 mM, Figure 11Bc). The RD duration under this condition was 235.9 ± 53.5 ms (Figure 11Be, Ca^++^ + Aβ_1–42_, *n* = 9) and was significantly shorter (*p* < 0.05, unpaired Student’s *t*-test) than the RD duration evoked under the control conditions in the absence of Ca^++^ and any other biologically active compounds (564.3 ± 39.6 ms, *n* = 192). At higher concentrations of Ca^++^ in the bath, RD was not evoked, despite the presence of Aβ_1–42_ in the pipette solution. As a control for the effect of Aβ_1–42_, the normal IgG antibody (4 μg/ml) was applied to the recording pipette solution. After 60 min of cell “dialysis” with IgG in the presence of Ca^++^ (0.3 mM) in the bath, RD was not evoked (Figures 11Ba,d,e, Ca^++^ + IgG, *n* = 6, *p* = 0.01, unpaired Student’s *t*-test).

### Repetitive Depolarizations Evoked During the RD Plateau

In the presence of Ca^++^ and the absence of TTX in the extracellular solution, typical action potentials were evoked during the current steps (Figure 2B). In the presence of TTX (0.5 or 1.0 or 10.0 μM) and the absence of Ca^++^, small-amplitude broad single (for example Figures 4D, 10Cc, 11Ac,Bc) or repetitive (spikelets, for example Figures 1Ab, 4B, 5Ac, 6Bb,c, 8Ba,b, 10Ac) depolarizations were observed at the beginning of RD (compare with Deisz, 1996). When Na^+^ was replaced with choline-Cl in the extracellular solution, TTX-resistant repetitive depolarizations were not evoked, irrespective of the 200-ms current step amplitude (Figure 4C). Nav1.5 and Nav1.8 are TTX-resistant and voltage-dependent Na^+^ currents that might potentially be responsible for these repetitive depolarizations. Nav1.5 channel currents can be blocked by high concentrations of TTX (Maier et al., 2003; Catterall et al., 2005). Nav1.8 channel currents are blocked by A803467 (Jarvis et al., 2007), A887826 (Zhang et al., 2010), or PF-01247324 (Payne et al., 2015). Bath application of TTX (10 μM), A803467 (1 μM), A887826 (10 μM), or PF-01247324 (3 μM) did not abolish either RD or the small-amplitude repetitive depolarizations evoked during RD (not shown). The properties of these TTX-resistant small repetitive depolarizations were not further investigated.

## Discussion

In this study, layer V mPFC pyramidal neurons were synaptically isolated due to the bath presence of GABAergic and glutamatergic transmission blockers and a voltage-gated Na^+^ channel blocker (TTX). In the tested neurons, two membrane currents were concomitantly activated after the hyperpolarizing step: a. a low-threshold, TTX-resistant inward Na^+^ current that evoked RD; and b. an outward K^+^ current through BK channels that opposes Na^+^-dependent depolarization. RD was observed after preventing BK channel activation. The RD amplitude was 30 mV above the resting membrane potential level (−68 mV), and it lasted 560 ms under our experimental conditions.

### Mechanism of Hyperpolarization Required to Evoke RD

Na^+^-dependent RD was evoked in pyramidal neurons after a hyperpolarizing voltage step below −80 mV, if BK channels were inhibited. Hyperpolarization of this magnitude was recorded in mPFC pyramidal neurons in experiments resembling physiological conditions, such as during *in vitro* membrane potential recordings with sharp microelectrodes (Lavin and Grace, 2001; O’Donnell, 2003; Valenti and Grace, 2009). Moreover, hyperpolarization below −80 mV can be elicited in these neurons by electrical stimulation of the entorhinal cortex (Valenti and Grace, 2009), locus coeruleus (Branchereau et al., 1996), or CA1 subiculum (Thierry et al., 2000) or through activation of serotoninergic receptors (Beique et al., 2004; Goodfellow et al., 2009). Marked hyperpolarization can also be evoked by GABA released from interneurons activated by acetylcholine (Aracri et al., 2010) or serotonin (Cozzi and Nichols, 1996; Abi-Saab et al., 1999).

### BK Channel Inhibition Is Required to Trigger RD

We obtained three lines of evidence indicating that RD can be evoked in the absence of outward K^+^ current activation through BK channels. (1) RD could be evoked after bath depletion of Ca^++^. The removal of Ca^++^ from the extracellular solution decreases the baseline Ca^++^ concentration in the cytoplasm of both neurons (Magee et al., 1996; Pinilla et al., 2005; Cheek and Thorn, 2006; Nichols et al., 2007) and nonneuronal cells (Mignen et al., 2017). Reducing the concentration of Ca^++^ in the cytoplasm leads to a marked increase in the voltage threshold of BK channels (Berkefeld and Fakler, 2013). (2) RD could be evoked in the presence of a selective BK channel blocker (paxilline) in the bath, despite the presence of Ca^++^ in the extracellular solution. (3) Finally, RD could be evoked when the tested cells were loaded with the Ca^++^ chelator BAPTA, despite the extracellular presence of Ca^++^. BAPTA eliminates free Ca^++^ from the cytoplasm and considerably elevates the voltage activation threshold of BK channels (Fakler and Adelman, 2008).

The activation of CaSR by Ca^++^ leads to the activation of BK channels (Vassilev et al., 1997; Chattopadhyay et al., 1999; Bandyopadhyay et al., 2007). Conversely, blockade of CaSR should lead to a decrease in BK channel activity. Indeed, we found that in the presence of a CaSR blocker, RD could be evoked, provided that the concentration of Ca^++^ in the extracellular solution did not exceed 0.1 mM. This finding supports the concept that activation of BK channels (in this case secondary to CaSR activation) is responsible for suppression of RD.

### BK Channel Inhibition Under Physiological and Pathophysiological Conditions

The activation of PKC inhibits BK channels (Shipston and Armstrong, 1996; Schubert and Nelson, 2001; Tian and Laychock, 2001; Kizub et al., 2010; Zhou et al., 2010; van Welie and du Lac, 2011). In our study, RD could be evoked during activation of PKC, despite the presence of Ca^++^ (1 mM) in the extracellular solution.

In our study, depletion of Ca^++^ from the extracellular solution was an effective means of enabling RD. RD was blocked in the presence of Ca^++^ in the bath at concentrations of 0.1 mM or higher. A decrease in the Ca^++^ concentration in the extracellular solution may be caused by displacement of Ca^++^ from the extracellular solution to the cytoplasm of glial cells and neurons.

The resting concentration of free Ca^++^ in the cerebrospinal fluid is 1.1 mM (Nilsson et al., 1993, 1996; Hartig et al., 2001). During neuronal activity, the concentration of Ca^++^ in the synaptic cleft drops to 0.3 mM (Vassilev et al., 1997; Egelman and Montague, 1998, 1999; Rusakov et al., 1998) and to 0.1 mM locally in the cerebrospinal fluid (Nicholson et al., 1978; Benninger et al., 1980; Heinemann and Pumain, 1980; Krnjević et al., 1982; Pumain et al., 1985). Theoretical calculations indicate that during neuronal activity, the Ca^++^ concentration in the cerebrospinal fluid decreases locally to 0 mM (Egelman and Montague, 1998, 1999). Low levels of Ca^++^ in the extracellular solution are maintained for several seconds due to the relatively small volume of the cerebrospinal fluid in relation to total brain volume (less than 20%; Rusakov et al., 1998) and due to the very slow diffusion of Ca^++^ in the cerebrospinal fluid (Kullmann et al., 1999). Therefore, in theory, a Ca^++^ level of zero can be attained locally in the cerebrospinal fluid, thus enabling RD. The results of our study also indicate that incomplete depletion of Ca^++^ from the extracellular solution together with additional manipulations, such as the intracellular presence Aβ_1–42_, can also promote the emergence of RD.

### Mechanism of BK Channel Activation Following Hyperpolarization

RD was not visible at physiological Ca^++^ concentrations in the extracellular solution due to concomitant activation of the outward K^+^ current through BK channels, raising the question of how BK channels are activated following the hyperpolarizing voltage step to oppose the Na^+^ inward current and suppress RD. At resting Ca^++^ levels in the cytoplasm (~100–200 nM, for a review, Clapham, 2007), the voltage threshold of BK channels is in the range of the positive membrane potential and well above the resting membrane potential. Therefore, at rest, BK channels are not constitutively active in pyramidal neurons (Bock and Stuart, 2016). This result was confirmed in our study because bath application of paxilline did not evoke depolarization in the tested pyramidal neurons, suggesting the absence of a constitutive outward K^+^ current through BK channels.

In the presence of the unselective voltage-gated Ca^++^ blocker Cd^++^ (Neumaier et al., 2015), RD could occur, despite the presence of Ca^++^ at a physiological concentration (1 mM) in the bath. This result suggests that an inward Ca^++^ current through voltage-dependent channels is responsible for BK channel activation and RD suppression. Two low-threshold and voltage-dependent Ca^++^ currents that may be activated when the hyperpolarizing voltage step returns to the resting membrane potential level have been described: T-type Ca^++^ currents and the recently described low-threshold L-type Ca^++^ currents.

Layer V cortical neurons express low-threshold, voltage-dependent, fast-inactivating T-type Ca^++^ channels (Sayer et al., 1990; de la Peña and Geijo-Barrientos, 1996; Craig et al., 1999; McKay et al., 2006). T-type channels are functionally (Smith et al., 2002) and structurally (Engbers et al., 2013b; Rehak et al., 2013) coupled to BK channels (Turner and Zamponi, 2014) and are largely inactivated at the resting membrane potential. Therefore, membrane hyperpolarization and subsequent depolarization could de-inactivate T-type channels (Molineux et al., 2006; Zamponi et al., 2010). Additionally, slowly inactivating, low-threshold L-type Ca^++^ channels (Magee et al., 1996; Lipscombe et al., 2004; Navedo et al., 2005; Kolaj et al., 2016), which form complexes with BK channels (Grunnet and Kaufmann, 2004; Guéguinou et al., 2014), have been described in pyramidal neurons. Our data indicate that at Ca^++^ concentrations of 0.1 mM in the bath, RD cannot be evoked. However, after separate or simultaneous blockade of L- and T-type Ca^++^ channels, RD is evoked, despite the presence of 0.1 mM of Ca^++^ in the extracellular solution. However, RD could not be evoked, despite the presence of L- and T-type Ca^++^ channel blockers at higher Ca^++^ concentrations in the extracellular solution.

Altogether, our results indicate that physiological concentrations of Ca^++^ in the extracellular solution have an inhibitory effect on RD, which was completely abolished in the presence of Cd^++^ in the bath and only partially eliminated after blockade of T- and L-type channels. Therefore, in addition to T- and L-type channels, Ca^++^ likely enters the cytoplasm through as yet undefined low-threshold and voltage-dependent Ca^++^ channels after the hyperpolarizing voltage step.

### Mechanism of the Plateau-Like Depolarization During RD

The inward Na^+^ current was responsible for RD because the latter was abolished after Na^+^ depletion from the extracellular solution. The involvement of HCN and NALCN channels, which are also permeable to Na^+^, was excluded as the source of the inward Na^+^ current.

The following features suggest that activation of Nav1.9 channels might be responsible for the inward Na^+^ current and RD in the tested neurons. (1) The effector responsible for RD was permeable to Na^+^. (2) RD was blocked by the presence of an anti-Nav1.9 antibody in the intracellular solution. (3) Similar to the Nav1.9 channel currents in neurons of the dorsal root ganglia (DRG; Coste et al., 2004; Dib-Hajj and Waxman, 2015) and mPFC pyramidal neurons (Gawlak et al., 2017), RD was resistant to TTX. (4) The voltage threshold of RD was close to the resting membrane potential, similar to Nav1.9 channel currents in DRG neurons (Coste et al., 2004; Dib-Hajj and Waxman, 2015) and mPFC pyramidal neurons (Gawlak et al., 2017). (5) The RD threshold was reduced in the presence of F^−^, similar to the activation threshold of Nav1.9 channels in DRG neurons (Coste et al., 2004). (6) Steady-state inactivation of RD was removed by membrane hyperpolarization to −80 mV, similar to the behavior of Nav1.9 currents, which were de-inactivated at −80 mV in DRG neurons (Coste et al., 2004; Dib-Hajj and Waxman, 2015) and in mPFC pyramidal neurons (Gawlak et al., 2017). (7) It was recently demonstrated that Nav1.9 channels are present in the mPFC (Gawlak et al., 2017; Radzicki et al., 2017), including in layer V pyramidal neurons (Kurowski et al., 2015). (8) Nav1.9 channels undergo slow inactivation, which is removed by cell membrane hyperpolarization (Rugiero et al., 2003; Coste et al., 2004; Maingret et al., 2008; Lin et al., 2016). This finding agrees with the assumption that Nav1.9 channel current is responsible for RD because RD could be evoked only after a prior hyperpolarizing step below −81 mV.

mPFC pyramidal neurons projecting to subcortical structures show small RD after a hyperpolarizing current step. In pyramidal neurons projecting to the cortex, RD was not found. The RD described by others depended on Na^+^ current flowing through HCN channels (Dembrow et al., 2010; Gee et al., 2012), while the RD described in our study had a much larger amplitude (30 mV) than the RD described in other studies (<1 mV) and depended on the activation of TTX-resistant, low-threshold Na^+^ current, was not blocked by HCN channel blockers and was expressed in an overwhelming majority of tested pyramidal neurons. Therefore, it seems that the RD described by others was produced by different mechanisms than the RD depicted in our study.

### Significance of RD

1. The present study suggests that inhibition of BK channels due to PKC activation enabled Na^+^-dependent RD, despite the presence of a physiological concentration of Ca^++^ in the extracellular solution. PKC is an effector of numerous transduction systems controlled by metabotropic receptors (Katritch et al., 2013). This finding raises the possibility that activation of metabotropic receptors and PKC-linked transduction pathways enable RD.
2. Aβ_1–42_ accumulates intracellularly in the neurons of Alzheimer’s disease patients (Takahashi et al., 2002) and in the brains of Alzheimer’s disease model mice (Oddo et al., 2003; Billings et al., 2005). In our study, application of Aβ_1–42_ to the cytoplasm of pyramidal neurons enabled RD, despite the presence of 0.3 mM of Ca^++^ in the bath. This finding indicates the potential existence of positive feedback in brains with dementia. When the Ca^++^ concentration drops to 0.3 mM in the cerebrospinal fluid (due to an increase in neuronal activity), RD appears and further enhances neuronal activity. This phenomenon may explain the mechanism responsible for the observed increase in neuronal activity in patients with senile dementia (Ferreri et al., 2003).
3. Depletion of Ca^++^ from the extracellular solution occurs during rapid neuronal discharge in seizures (Yaari et al., 1983; Heinemann and Hamon, 1986; Han et al., 2015; Nardone et al., 2016). Additionally, the occurrence of seizures is supported by the decrease in Ca^++^ ion concentration in the cerebrospinal fluid (Yaari et al., 1983; Heinemann and Hamon, 1986; Han et al., 2015; Nardone et al., 2016), suggesting that in quiescent neurons, located close to the repetitively firing neurons, RD and activity increases can be evoked. The activity increases in quiescent neurons can be induced, providing that they are in the range of low Ca^++^ concentration in the cerebrospinal fluid and they receive inhibitory input. Moreover, our study indicates that increased activity in such neurons can be favored by either inhibition of T- or L-type Ca^++^ channels or by blockade of BK channels (e.g., by PKC activation), and can be prevented by inhibition of persistent and TTX-resistant inward Na^+^ current.
4. Up- and down-states are defined as intermittent shifts of membrane potential from hyperpolarization (down-state) to depolarization (up-state) in cortical and subcortical neurons (typically lasting a few 100 ms each, Wilson and Kawaguchi, 1996; McCormick et al., 2003). During down-state, the membrane potential can drop below −80 mV in mPFC pyramidal neurons (e.g., Figure 1 in Lewis and O’Donnell, 2000). Slow membrane potential oscillations in mPFC pyramidal neurons are thought to be dependent on recurrent networks of local neuronal circuits (McCormick et al., 2003), intrinsic neuronal properties (Seamans and Yang, 2004), or synaptic inputs into mPFC pyramidal neurons (O’Donnell, 2003). One may presume that low-threshold, TTX-resistant, and voltage-dependent inward Na^+^ current can be activated and can support the up-state when the membrane potential shifts from the down-state to the up-state. However, such reinforcement of the up-state can occur only if it is accompanied by inhibition of BK channels, such as during PKC activation or in the intracellular presence of Aβ_1–42_.
5. One feature of prefrontal cortex pyramidal neurons is the capacity to generate prolonged depolarizations with a series of action potentials at the depolarization peak (persistent activity) and lasting markedly longer than the triggering stimulus (for review, see Galloway et al., 2008; Riley and Constantinidis, 2016). Several mechanisms have been proposed to be responsible for the generation of the prolonged depolarization, related preferentially to the excitatory input to the mPFC pyramidal neurons. For example, it was demonstrated that depolarization may depend on the glutaminergic and dopaminergic input to pyramidal neurons from distant cells (Lewis and O’Donnell, 2000; Seamans et al., 2003). Moreover, it was indicated that prolonged depolarizations may be dependent on local circuits (McCormick et al., 2003; Haider et al., 2006) or intrinsic properties of these neurons (Zylberberg and Strowbridge, 2017). It is most likely that several mechanisms are responsible for prolonged depolarization and persistent activity. Our study shows that RD evoked after hyperpolarization resembles the prolonged depolarizations elicited by excitatory input to mPFC pyramidal cells in terms of amplitude, duration and the repetitive spikes seen during the depolarization plateau. However, the mechanisms of prolonged depolarizations described by others and of RD described in our study are triggered by different mechanisms (hyperpolarization vs. depolarization); moreover, the mechanisms of their creation are also different.

## Author Contributions

PK and PS conceived and designed the experiments, discussed the data and wrote the manuscript. PK performed the experiments, analyzed the data and prepared the figures. KG executed some experiments.

## Conflict of Interest Statement

The authors declare that the research was conducted in the absence of any commercial or financial relationships that could be construed as a potential conflict of interest.
